# Trans-scaphoid Lunate Dislocation Rare Injury: A Case Report

**DOI:** 10.7759/cureus.44072

**Published:** 2023-08-24

**Authors:** Dhafer Alshehri, Aishah Mousa, Basim Al Ahmadi

**Affiliations:** 1 Orthopaedic Surgery, Faculty of Medicine in Rabigh, King Abdulaziz University, Jeddah, SAU; 2 Orthopaedics, King Fahad Hospital, Jeddah, SAU; 3 Orthopaedic Surgery, King Fahad General Hospital, Jeddah, SAU

**Keywords:** carpal injury, wrist dislocation, wrist injury, lunate dislocation, lunate fracture dislocation, perilunate fracture

## Abstract

Lunate dislocations are complex and uncommon wrist injuries, often resulting from high-energy trauma like road traffic accidents, falls, and industrial mishaps. Timely diagnosis and appropriate surgical management are critical to achieving favorable outcomes and minimizing long-term disability. Here, we present a case of a 22-year-old male who sustained a trans-scaphoid lunate fracture dislocation due to a motorcycle accident. Prompt wrist x-ray evaluation and subsequent surgical interventions led to the successful restoration of wrist function and stability. This case report highlights the importance of early recognition and discusses the accurate diagnosis and timely management of lunate fracture dislocations to avoid long-term complications and disability.

## Introduction

Lunate fracture dislocation is a rare and complex wrist injury that results from high-energy trauma, such as a fall from a height or a road traffic accident [1]. This injury leads to a specific pattern of palpable wrist deformity due to the disruption of bones and ligaments, impacting hand function and stability [2]. This type of injury is estimated to occur in 10% of all wrist injuries, and its mechanism involves forced hyperextension, ulnar deviation, and intercarpal supination of the wrist [1,2]. The wrist joint is a complex structure that allows for a wide range of movements, making it susceptible to various injuries, including fractures and dislocations. A trans-scaphoid lunate fracture dislocation occurs when there is a combination of fractures involving the bones of the carpal region, along with the dislocation of the lunate bone relative to the radius [3]. The adjacent carpal bones typically dislocate dorsally, while the lunate bone remains in its normal position connected to the distal radius. However, in rare cases, the lunate bone may dislocate volarly into the space of Poirier [1,2].

Due to the rarity and complexity of this injury, timely and accurate diagnosis is crucial to prevent long-term complications and disability, such as median neuropathy, post-traumatic scapholunate advanced collapse (SLAC) wrist arthritis, chronic instability patterns, such as dorsiflexed intercalated segment instability (DISI), and volar intercalated segment instability (VISI), as well as delayed healing of fractures [4,5]. Computed tomography (CT) scans are particularly valuable as they offer a more comprehensive view of the injury's extent and assist in treatment planning [3]. Management of lunate fracture dislocation often requires prompt surgical intervention to restore the normal alignment of the wrist and stabilize the damaged structures [6]. Understanding the complexity of lunate fracture dislocation is critical for healthcare workers caring for patients with wrist injuries, as it will guide them in making informed decisions about the most effective therapy techniques. Here, we present a case of a lunate fracture dislocation in a young male adult and discuss the diagnosis and surgical management that led to a successful outcome.

## Case presentation

A 22-year-old man presented to our hospital one day following a motorcycle accident, complaining of right wrist swelling, pain, and abrasions. On physical examination, he was stable with normal vital signs. He had abrasions on the wrist and forearm, and his wrist was swollen and deformed with volar prominence, tenderness, and limited range of motion. The distal neurovascular bundle was intact.

**Figure 1:**
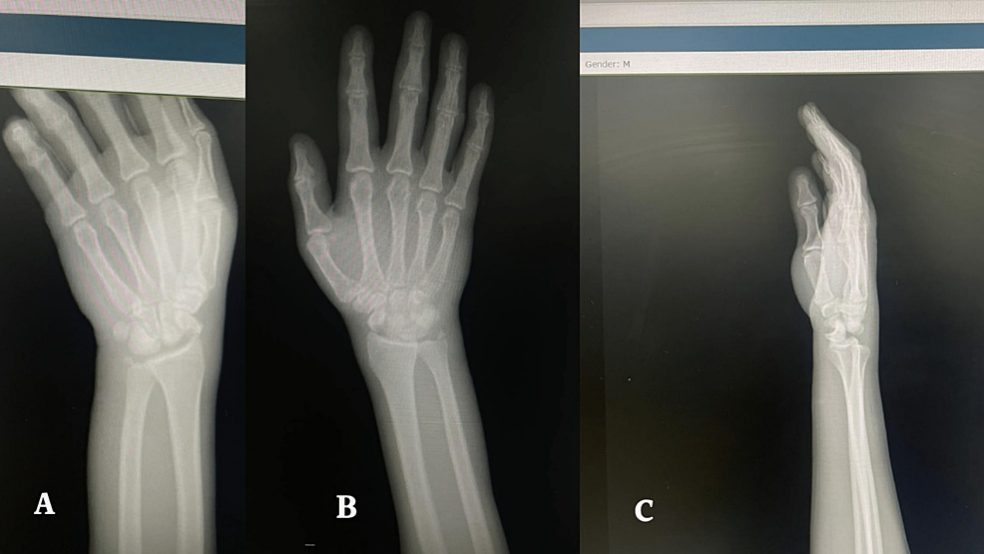
Postero-anterior (A), antero-posterior (B) and lateral views of the right wrist x-ray showing a lunate volar dislocation and scaphoid fracture

X-ray of the right wrist revealed a lunate dislocation, scaphoid fracture and radial styloid fracture (Figures 1A-1C). A right lunate volar dislocation and scaphoid fracture were diagnosed, and the patient was planned for open reduction and headless screw fixation of the scaphoid in addition to K wire fixation of the lunate dislocation.

**Figure 2:**
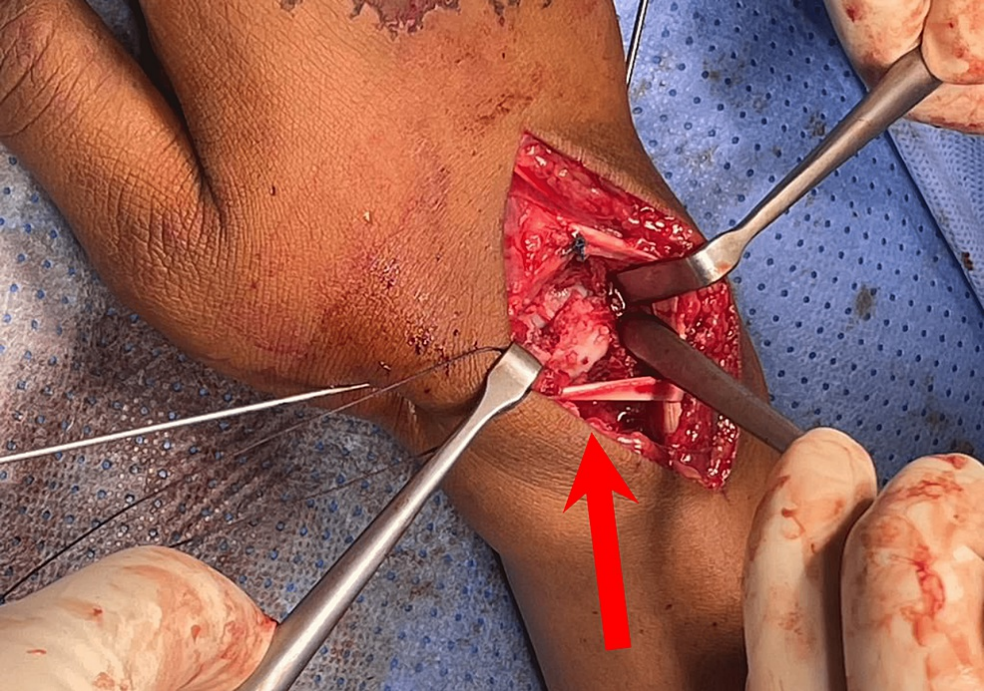
Dorsal surgical approach

Given the severity of the injury and the presence of associated fractures, urgent surgical intervention was planned. The patient was taken to the operating room. Under general anesthesia, we started with a volar approach and did carpal tunnel release, followed by a dorsal approach to access the carpal bones (Figure 2). Capsulotomy was done using a radial base flap. Intraoperative findings: scaphoid fracture with moderate dorsal comminution and intact scapholunate ligament; the lunate reduction was made via longitudinal traction and dorsal force application followed by scaphoid fracture reduction using K wires as a joystick and fixed with 1.4 K wires provisionally.

**Figure 3:**
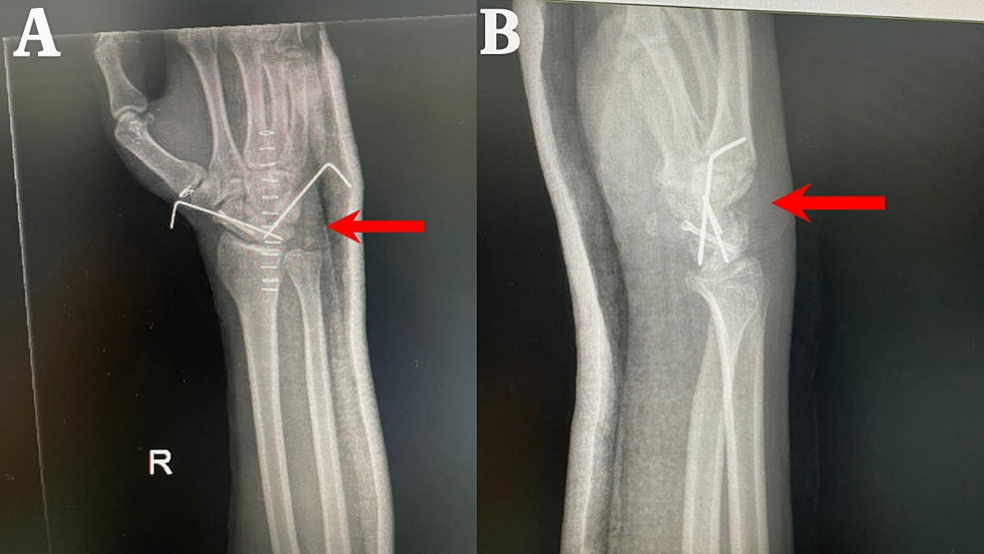
Postero-anterior (A) and lateral (B) views of post-operative x-ray showing Herbert screw and K wires in place

The scaphoid fracture was fixed with a 2.5 mm headless screw (Stryker) inserted from the volar to the dorsal side. A 1.2 K wire was used to stabilize the scapholunate, and a 1.4 K wire was used to stabilize the lunotriquetral under c-arm guidance. We did wound irrigation and closure in layers, including the dorsal capsular flap (Figures 3A, 3B). A sterile dressing and above elbow back slab were applied.

Postoperatively, the patient was seen for the first time at two weeks to check the wounds and to remove the sutures. At six weeks postoperatively, K wires were removed, and he was referred to physiotherapy to start range of motion exercises.

**Figure 4:**
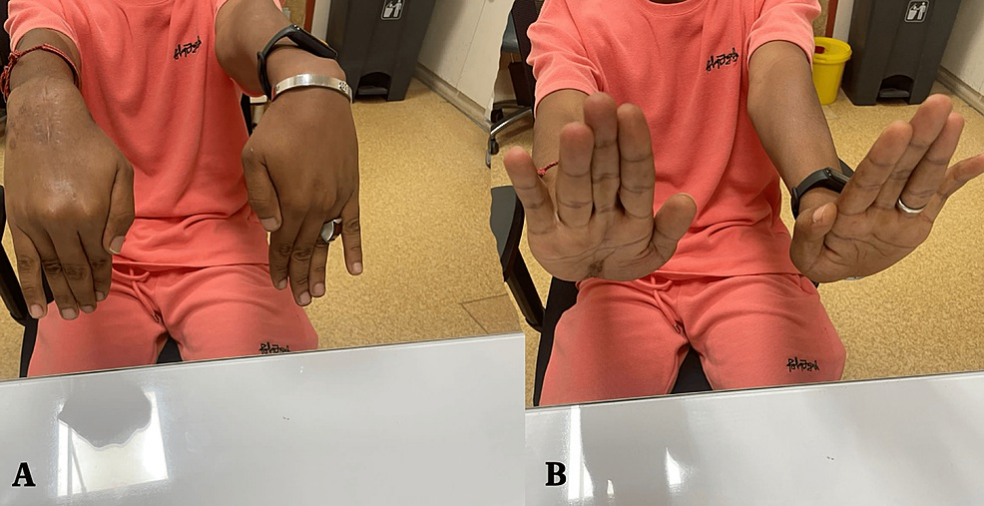
Two months post-operation with full recovery of the hand's range of motion with dorsal (A) and volar (B) wounds fully healed

Regular follow-up visits and radiographs were conducted to monitor the progress of bone healing and the maintenance of reduction, and the patient demonstrated improvement in wrist function and range of motion (Figures 4A, 4B), with good bone healing and proper alignment of the carpal bones.

## Discussion

Perilunate fracture dislocations are rare but severe injuries requiring a high suspicion level for accurate diagnosis. They present a significant challenge for clinicians due to their potential for long-term disability and complications if not diagnosed and treated promptly [7]. Timely recognition of the injury through radiographic images, followed by early surgical intervention, is crucial in achieving successful outcomes and preventing long-term complications [2,4,7]. Perilunate fracture dislocations are missed in 25% of cases at the initial presentation [8]. This type of fracture results from high-energy traumas, such as road traffic accidents, industrial mishaps, and falling from a height, with hyperextended wrists, and as for our patient, the clinical presentation involves a grossly deformed wrist with tenderness on palpation and a limited range of movement [2]. Couples with a likelihood of missed diagnoses, this clinical presentation highlights the importance of clinical suspicion for lunate dislocation in patients with high-energy trauma and wrist injuries. Though wrist x-ray revealed lunate dislocation and scaphoid fracture in our case, standard radiographs may reveal gross deformities but can miss associated fractures and ligamentous injuries. Therefore, a CT scan with coronal, sagittal, and 3D reformatted images might be required when radiographic examination does not adequately show the full scope of wrist injuries [3] since the CT images can clearly reveal associated fractures of the scaphoid and triquetrum bones aiding in surgical planning and ensuring appropriate management [9].

The treatment approach involves either open reduction and internal fixation (ORIF) or closed reduction and internal fixation (CRIF), followed by at least four weeks of immobilization [3,10]. Studies showed that CRIF leads to better outcomes than ORIF, making it preferred over ORIF [10]. The surgical method used in the presented case was a double approach with ORIF, which gives adequate exposure to address all the injuries [11]. The use of screws and Kirschner wires to fix the fracture and maintain the reduction is a popular method. Ligament restoration is critical for restoring wrist stability and preventing chronic instability. Fortunately, we had no ligamentous injuries in this case [8,11]. Scapholunate interosseous and lunotriquetral ligaments were intact in our case. In case of injuries to these ligaments, DISI and VISI can ensue, and Magnetic Resonance Imaging (MRI) helps detect such injuries [3,4,10]. The preferred surgical approach for lunate fracture dislocation is an open reduction and repairing the ligaments alongside percutaneous pin fixation. When this dislocation is accompanied by a fracture of the distal radial styloid or a carpal bone, internal fixation is the favored option [7].

Following surgery, the patient's wrist was immobilized with a back slab to allow bone healing and proper alignment of the carpal bones, and regular follow-up visits showed good results in terms of range of motion. Perilunate fracture dislocations are relatively rare, and their treatment necessitates a team effort comprising orthopedic surgeons, radiologists, and physiotherapists. The presented case emphasizes the importance of maintaining a high index of suspicion in cases of high-energy trauma and wrist injuries. Furthermore, the case emphasizes the significance of early diagnosis and surgical intervention to restore carpal stability, avoid long-term problems, and enhance patient outcomes [4,7]. Delayed treatment may result in post-traumatic wrist arthritis, chronic instability, and delayed fracture union, all of which negatively influence the patient's quality of life. This is supported by research showing that delayed treatment leads to inferior outcomes compared to immediate intervention [10]. Therefore, when the facilities are not equipped to manage this type of fracture, early referral to specialized treatment facilities for perilunate injuries enables timely management with optimal outcomes.

## Conclusions

The case described a rare wrist injury, with successful restoration of function and stability due to prompt diagnosis, surgery, and postoperative therapy. This case also highlights the need for healthcare personnel to maintain a high index of suspicion for perilunate fracture dislocation in high-energy trauma, as a missed diagnosis can lead to debilitating chronic symptoms of instability and osteoarthritis.
